# Mental, Physical and Socio-Economic Status of Adults Living in Spain during the Late Stages of the State of Emergency Caused by COVID-19

**DOI:** 10.3390/ijerph19020854

**Published:** 2022-01-13

**Authors:** Elisabet Sánchez-Rodríguez, Alexandra Ferreira-Valente, Filipa Pimenta, Antonella Ciaramella, Jordi Miró

**Affiliations:** 1 Universitat Rovira i Virgili, Department of Psychology, Research Center for Behavior Assessment (CRAMC), Unit for the Study and Treatment of Pain—ALGOS, 43007 Tarragona, Catalonia, Spain; elisabet.sanchez@urv.cat; 2 Institut d’Investigació Sanitària Pere Virgili, 43007 Tarragona, Catalonia, Spain; 3 William James Center for Research, ISPA—University Institute, 1100-304 Lisbon, Portugal; mafvalente@gmail.com (A.F.-V.); filipa_pimenta@ispa.pt (F.P.); 4 Department of Rehabilitation Medicine, University of Washington, Seattle, WA 98195, USA; 5 Lab. of Psychosomatic, GIFT Institute of Integrative Medicine, 56126 Pisa, Italy; ciarantogift@gmail.com

**Keywords:** COVID-19, confinement, mental health, physical health, socio-economic, Spain

## Abstract

Research has shown that the confinement measures implemented to curb the spread of COVID-19 can have negative effects on people’s lives at multiple levels. The objective of this cross-sectional study was to better understand the mental, physical, and socio-economic status of adults living in Spain during the late stages of the state of emergency caused by COVID-19. Five hundred and forty-four individuals responded to an online survey between 3 June and 30 July 2020. They were asked to report data about their mental and physical health, financial situation, and satisfaction with the information received about the pandemic. Means, percentages, *t*-test, ANOVAs, and logistic regressions were computed. A third of the participants reported symptoms of anxiety, depression, and stress, and worries about their health and the future. Participants also described mild levels of fatigue and pain during lockdown (66%), and a reduction in household income (39%). Respondents that were female, younger, single, and with lower levels of education reported experiencing a greater impact of the COVID-19 pandemic. The data showed that the negative effects of lockdown were present in the late stages of the state of emergency. The findings can be used to contribute to the development of programs to prevent or mitigate the negative impact of confinement measures.

## 1. Introduction

The world is currently experiencing an extraordinary health and economic crisis due to COVID-19. The Severe Acute Respiratory Syndrome Coronavirus 2 (SARS-CoV-2) virus began to spread at the end of 2019 in China [1] and, in a short time, it expanded worldwide, causing unprecedented restrictions in most countries: border closures, lockdowns, social distancing, etc. Social distancing and confinement measures to control the spread of the disease had previously been implemented, for example, in Asia in 2003 in relation to the first signs of the SARS infection, and in Africa in 2014 during the Ebola crisis [2,3], but never at the present scale.

Research has shown that confinement measures, although necessary for effectively controlling disease, are associated with problems at multiple levels: mental, physical and socio-economic. For example, Lee et al. [4] found that the most prevalent symptoms among people who were confined during the SARS epidemic included low mood (reported by 73% of those surveyed), irritability (57%), and insomnia (34%). These symptoms persisted over time and led to avoidance behaviors once lockdown was lifted, such as avoiding contact with other people, avoiding closed places and/or all public spaces in general, thus increasing their negative impact. Importantly, studies have shown that the psychological consequences of confinement can continue for several years after the situation that caused it is resolved [5]. For example, Wu et al. [6], in a study with 549 healthcare professionals, found that participants reported symptoms of post-traumatic stress for up to three years after SARS-related confinement. A recent systematic review [7] summarized the consequences that being in lockdown has on individuals and found significant negative psychological effects, particularly in relation to emotional state (e.g., greater symptoms of post-traumatic stress, fear, confusion, anger, and frustration). Similarly, Xiong and colleagues [8] have reported that the COVID-19 pandemic has caused high levels of anxiety, depression, and stress across many different countries. This systematic review identified the following risk factors for greater negative effects to mental health: being female, young (up to 40 years old), having a physical or psychiatric illness, being unemployed, and being continuously exposed to information about COVID-19.

Confinement measures have also been associated with physical symptoms such as pain, fatigue, and insomnia [9,10]. For example, Toprak et al. [11] found that individuals who stayed at home during lockdown reported higher levels of lower back pain than those who continued working outside the home. Finally, confinement prevented many people from engaging in their regular professional activities, potentially leading to serious socio-economic problems. These types of problem, in turn, can result in significant psychological disorders, such as anger and anxiety, months after the end of lockdown [12,13,14]. For example, in a sample of 369 adults from China, Zhang et al. [14] found that 25% of them had to stop working during lockdown, and that this situation was associated with worse mental and physical health.

There is mounting evidence regarding the stressors, both during and after lockdown, that could predict the psychological impact of confinement [7]. During confinement, the following stressors have been identified: duration of confinement, fear of being infected or possibly infecting others, disruption of routines, reduced physical and social contact, frustration, not having guaranteed basic supplies (e.g., food, water, medical care), not having enough information, loneliness, and homeschooling [5,15,16,17,18,19]. In the post-confinement period, studies have found that financial difficulties are the main source of stress [7]. In addition, research has shown that individuals with previous physical symptoms, such as pain, may be more vulnerable to the impact of confinement, both during and afterwards. For example, Wang et al. [20] found that individuals with chronic physical pathologies, who rated their health status as poor, also had higher levels of stress, anxiety, and depression during the first phase of the COVID-19 epidemic in China.

There are few studies on the effects that a crisis of this magnitude can have on the mental, physical, and socio-economic status of individuals living in Spain. Taken as a whole, they show that a significant number of adults report symptoms of depression (between 19 and 41%, depending on the study [21,22]), anxiety (approximately 25% [21,22]), stress (41% [22]), and post-traumatic stress syndrome (16% [21]). Interestingly, studies have revealed that socio-demographic variables like sex, employment status, and education are significantly related to the development of psychological distress. For example, Esteban-Gonzalo et al. [23], in a study of 801 adults, found that being young, female, single, unemployed, and with only a basic education was significantly associated with the development of psychological distress.

To the best of our knowledge, all the studies on the impact of confinement measures in Spain have focused on the initial stage of the state of emergency (i.e., the strict shelter-in-place phase during the first few weeks of lockdown in March and April 2020). However, research has shown that the impact of these measures increases as the duration of the confinement increases [7]. Therefore, in order to be able to develop specific programs to mitigate and manage the long-term mental, physical, and socio-economic consequences of confinement, it is essential to determine the impact that the later stages of lockdown have on the lives of individuals living in Spain. Furthermore, many studies have been conducted with a relatively narrow perspective; that is to say, they have focused on one or two domains, such as the impact of confinement on mental health (e.g., [21]). However, the impact of lockdown extends to other domains, affecting physical and socio-economic well-being which, in fact, are interrelated [9,10,14]. Information about the impact of lockdown in all domains is of key importance to help prevent or reduce these effects in future similar situations. Therefore, the main aim of this study was to expand the body of knowledge regarding the mental, physical, and socio-economic status of individuals living in Spain in the late stages of the state of emergency and lockdown caused by COVID-19. Specifically, we sought to determine the (1) mental health (i.e., levels of anxiety, depression, stress, and worry), (2) physical health (i.e., pain, fatigue, physical exercise, overall health, and quality of life), and (3) socio-economic (i.e., household income and interference in daily life) conditions of people living in Spain during this period. Furthermore, we studied whether these variables were related to age, gender, education, and marital status. We also studied the opinions of the participants about the handling of the pandemic in Spain (i.e., satisfaction with the information received, satisfaction with the handling of the situation by the competent authorities, and compliance with confinement measures).

## 2. Materials and Methods

### 2.1. Participants

The study sample included 544 participants. The inclusion criteria for the study were: (1) being 18 years old or older; (2) being able to read and write Spanish; (3) living in Spain during lockdown; (4) having access to the Internet; and (5) providing informed consent.

### 2.2. Procedure

This study was approved by the Ethics Committee for Medical Research of the Pere Virgili Health Research Institute (ref. 117/2020). To recruit the participants, we distributed a link to our online survey via our personal and research group social media (i.e., Facebook, Whastapp, Instagram, Twitter) and via institutional sites (i.e., University webpage, mails to the university community). (http://algos-dpsico.urv.cat/survey/index.php/654243?lang=es, access on 28 December 2021). A non-probabilistic snowball approach was used (i.e., we asked potential participants to distribute the survey information among their friends, family and colleagues). First, when potential participants accessed the survey, they found a detailed explanation of the study and the informed consent page. Then, in order to participate, those interested had to sign the consent page. The online survey was available for participation from 3 June to 30 July 2020.

### 2.3. Measures

Demographics: Participants reported data about their age, gender, education, province of residence, marital status, and monthly household income.

Mental health: We used the Spanish version of the 21-item Depression, Anxiety and Stress Scale to measure symptoms of anxiety, depression, and stress (DASS-21 [24,25]). Respondents were asked to rate the degree to which they had experienced each symptom during confinement on a 4-point Likert-type scale, from 0 (“Did not apply to me at all”) to 3 (“Applied to me very much or most of the time”). The total score of each subscale is the sum of the responses multiplied by two. The scores for each subscale can be classified according to their severity as follows: for anxiety: normal (0–7), mild (8–9), moderate (10–14), severe (15–19), and extremely severe (≥20); for depression: normal (0–9), mild (10–13), moderate (14–20), severe (21–27), and extremely severe (≥28); and for stress: normal (0–14), mild (15–18), moderate (19–25), severe (26–33), and extremely severe (≥34). In this study, the internal consistency was good for the subscales of anxiety and depression (α = 0.83 and 0.89, respectively) and excellent for the subscale of stress (α = 0.90). In addition, participants were asked about their worries during the COVID-19 pandemic related to the following issues: health, food, employment, being able to pay mortgage or rent, being able to pay bills, income, future, education, family relationships, solidarity, and an “other” category) using a 0–10 numerical rating scale in which 0 meant “Not at all worried” and 10 “Completely worried”.

Physical health: Participants were asked about the following issues: pain, fatigue, physical exercise, overall health, and quality of life. First, they reported whether they experienced pain during lockdown. Then, we used the Silhouettes of Fatigue Scale (SFS [26]) to assess the level of fatigue they experienced during confinement. The SFS consists of six human silhouettes that show an increasing level of fatigue from left to right. The leftmost figure represents “No fatigue” and each additional figure to the right represents gradually higher levels of fatigue up to the rightmost figure, which represents “A lot of fatigue”. The SFS has been shown to provide valid data when used in adults [26]. Participants also provided data about physical exercise during lockdown; they had to respond to the following question: “During the state of emergency, have you been able to do any kind of physical exercise? If so, what type of exercise?” We also asked participants to report their subjective overall health and quality of life using two single items from the 2010–2012 World Values Survey (Health Perception) [27]. Participants were asked to respond using the following scale: 1 = “Excellent”, 2 = “Very good”, 3 = “Good”, 4 = “Reasonable”, and 5 = “Bad”.

Socio-economic information: Participants reported data about changes in their monthly household incomes and about the interference of COVID-19 with specific areas of their daily life: life in general, interpersonal relationships, work, motivation to work, satisfaction with life, and happiness. Participants reported the degree to which the COVID-19 pandemic interfered in each area of their life using a numerical rating scale with the following anchors: 0 meaning “It does not interfere” and 10 meaning “It completely interferes”.

Information and handling of the COVID-19 pandemic: Participants were asked to respond to the following questions: (1) “Have you received enough information about the pandemic and the preventive measures in place?”, and (2) “What do you think about the government’s handling of the pandemic situation?” (in relation to this second question, participants were asked to report whether the situation could have been handled better and, if so, in what areas, and how); and (3) “To what extent do you trust the national healthcare system to respond to the pandemic?” Participants had to respond to this question using a 5-point numerical rating scale with anchors from 1 (“I have no confidence”) to 5 (“I have full confidence”).

### 2.4. Data Analyses

First, we computed the descriptive statistics (means and standard deviations for continuous variables, numbers and percentages for dichotomous variables) to describe the sample of participants and the study variables. Then, to test whether mental health (i.e., depression, anxiety and stress symptoms, and worries), physical health (i.e., fatigue, overall health, and quality of life), and socioeconomic impact (i.e., COVID-19 financial interference) were associated with gender, age, education, and marital status, we performed a series of independent *t*-tests (when addressing gender comparisons) and a series of one-way ANOVAs. When addressing comparisons regarding age, we considered youth (from 18 to 24 years old), early adulthood (from 25 to 40 years old), middle adulthood (from 41 to 65 years old), and older (over 65 years old); educational levels were categorized as no studies or only elementary education, secondary education, or university degree; and marital status was classified as single, married or living with a partner, divorced, or widow/widower. Finally, we conducted three logistic regression analyses to further study the association between socio-demographic (i.e., age, gender, marital status, and education) and socio-economic (i.e., change in household income) variables with mental health (i.e., anxiety, depression, and stress) variables. We evaluated the suitability of the data for the planned regression analysis by examining the independence of the variables and their linearity to the log odds). Anxiety, depression, and stress were the criterion variables and were dichotomized as “normal anxiety/depression/stress” vs. “significant levels of anxiety/depression/stress.

All the analyses were performed using the Statistical Package for Social Sciences for Windows version 25.0 (SPSS Inc., Chicago, IL, USA). Alpha was set at 0.05 for all analyses.

## 3. Results

### 3.1. Description of the Study Sample

The sample included 544 participants with an average age of 38.57 (SD = 14.99), most of whom were females (78%) in middle adulthood (41%). This was a fairly well-educated sample of individuals, as most of the participants (59%) reported having a university degree. In addition, most participants were married or living with their partner (52%). This sample included participants from all economic classes; however, most were clustered in the middle- and upper-class economic groups. Participants were from all Spanish autonomous communities; however, most of them were from the provinces of Tarragona (61%), Barcelona (11%), and the Balearic Islands (6%). See Table 1 for additional details.

### 3.2. Mental Health

Four hundred and sixty participants provided information about their anxiety, depression and stress symptoms, and worries. Almost a third of participants reported some anxiety symptoms, of whom 10% reported “severe” or “extremely severe” levels of anxiety. Similarly, a third also reported some symptoms of depression, with 10% of those being “severe” or “extremely severe”. Finally, another third of participants reported some stress, with 11% suffering from “severe” or “extremely severe” levels of stress. See Table 2 for detailed information.

Female participants reported significantly more symptoms of anxiety, depression, and stress than male participants did. The data showed that younger participants (<40 years old) reported significantly more symptoms of anxiety, depression, and stress than participants over 40. Moreover, participants with a university degree reported significantly fewer symptoms of anxiety, depression, and stress than participants with only a secondary education. Marital status also showed some significant associations with these variables. In particular, the data showed that single participants reported more symptoms of anxiety, depression, and stress than those married/living with a partner or divorced. See Table S1 for additional information.

The logistic regression model for anxiety was significant (X^2^ = 34.50, *p* < 0.001) and explained the 11% variance (R^2^ Nagelkerke = 0.112). Only age and gender emerged as significant, thus showing that females were 2.8 times more likely to report anxiety problems than males, and that younger participants were two times more likely to report anxiety problems than 25–40-year-olds, 2.6 times more likely than 41–65-year-olds, and 4.2 times more likely than over-65-year-olds. The value of the Hosmer-Lemeshow showed a good fit of the model (X^2^ = 3.66, *p*= 0.886; see Table 3).

The logistic regression model for depression was significant (X^2^ = 36.85, *p* < 0.001) and explained the 12% variance (R^2^ Nagelkerke = 0.119). Results showed that females were 1.9 times more likely to report depression symptoms than males and that singles were 2.1 times more likely to report depression symptoms than those married or living with their partner, and 3.5 times more likely than divorcees. The value of the Hosmer-Lemeshow showed a good fit of the model (X^2^ = 8.84, *p* = 0.356; see Table 3).

The logistic regression model for stress was significant (X^2^ = 32.93, *p* < 0.001) and explained a 11% of the variance (R^2^ Nagelkerke = 0.105). Results showed that age and gender emerged as significant showing that females were 3.3 times more likely to report depression symptoms than males. Although age emerged as significant, we did not find statistically significant differences between groups. The value of the Hosmer-Lemeshow showed a good fit of the model (X^2^ = 9.44, *p* = 0.306; see Table 3).

The participants in this study reported having different types of worries. The most prevalent ones were, in order of importance, related to health, the future, and support for and from others (see Table 2). Female participants worried significantly more than male participants across almost all issues. Interestingly, the statistics show that the youngest women worried significantly more about the future than older participants did, whereas those in early adulthood worried significantly more about work. Participants under 40 years old were significantly more worried than individuals over 40 about household income and paying the mortgage/rent. The statistics also showed that participants with university degrees reported being significantly less worried about food, household income, paying mortgage/rent/bills, and education than participants with only secondary educations did. Participants that were married or living with a partner reported significantly more worries about being supportive of others than single participants did. See Table S2 for additional details.

### 3.3. Physical Health

Three hundred and sixty participants (66%) reported having experienced pain during lockdown and, of these, almost half (48%) reported that pain was present for at least half of the days during the previous three months. The most frequent pain described was back pain (64%). Participants reported a mild level of fatigue (mean = 3.17, SD = 2.52), which statistics showed to be significantly higher among female participants. Interestingly, the majority of participants (75%) reported having practiced some type of physical exercise during lockdown. See Table 4 for detailed information.

Most participants (81%) reported good to excellent overall health, although 5% reported being in bad health (see Table 4). The statistics also showed that age and gender were significantly associated with overall health, with males and participants over 40 years old reporting worse overall perceived health. No statistically significant differences emerged in overall health related to education or marital status. See Table S3 for additional information.

The majority (80%) of the sample reported having at least a good quality of life, although 4% reported having a poor quality of life (see Table 4). Male participants reported significantly worse levels of quality of life than female participants. No significant differences emerged in quality of life related to age, education, or marital status. See Table S3 for additional information.

### 3.4. Socio-Economic Impact

Most participants reported no changes in their family income level (58%). However, 39% of the participants reported some degree of reduction in their household income. See Table 5 for additional information.

Participants also reported that the COVID-19 pandemic interfered moderately to highly in almost all areas of daily life. The domain in which the COVID-19 pandemic interfered the most was “life in general”, followed by “interpersonal relationships”, and “work”. See Table 5 for additional information.

There were statistically significant gender differences in COVID-19-related interference in the participants’ “motivation to work”, “satisfaction with life”, and “happiness”, with female participants reporting greater interference. We also found statistically significant associations between age and interference. Specifically, younger participants reported higher interference in “motivation to work” but lower interference in “interpersonal relationships” when compared with participants in the middle-aged group. Conversely, older participants reported significantly less interference than the other age groups in “life in general”, “work”, and “motivation to work”. Finally, single participants reported significantly higher interference in “motivation to work” than participants who were married/living with a partner. See Table S4 for additional information.

### 3.5. Information and Handling of the COVID-19 Pandemic

Almost half of the participants (47%) reported that they did not receive sufficient information about the pandemic situation. The issues in which information was missing the most were about “how the government was handling the pandemic”, “treatment for COVID-19“, and “the effects of COVID-19”. See Table 6 for additional information.

Most participants (91%) perceived that the government’s handling of the pandemic could have been better in Spain, and identified the following areas for improvement as the most important: “conducting mass COVID-19 testing on the population” and “advancing lockdown”. See Table 6 for additional information.

Finally, participants reported a medium to high level of trust in the Spanish health system to respond to the pandemic, and 97% reported having complied with governmental requests for sheltering in place.

## 4. Discussion

The objective of this study was to better understand the impact of the COVID-19 lockdown on the mental, physical, and socio-economic status of adults living in Spain in the late stages of the state of emergency and shelter-in-place order (i.e., 3 June to 30 July 2020).

In relation to the mental health status of participants, the data showed that a third of them reported anxiety, depression, and stress symptoms. Furthermore, 10% reported that these symptoms were severe or extremely severe. The most important worries reported by this sample were related to health and the future. Previous studies conducted in Spain during the early stages of the state of emergency and lockdown found that participants reported fewer symptoms of anxiety [22,23], and fewer symptoms of depression and stress [21]. It is unclear whether these results indicate that the impact increases with time or whether they are related to the specific characteristics of the samples, as we do not have the data to respond to this question. However, they do show that the impact of lockdown endures over long periods of time.

Young (under 40 years old), female, single, and less educated participants reported a greater impact on mental health (i.e., anxiety, depression, and stress symptoms). The data showed that females were 2.8 times more likely to report anxiety problems, 1.9 times more likely to report symptoms of depression, and 3.3 times more likely to report stress than males. Furthermore, singles were 2.1 times more likely to report symptoms of depression than those that were married or living with their partner, and 3.5 times more likely than divorced participants. Importantly, these findings are similar to those from previous studies with different samples (e.g., [8,22,28]), and may indicate that these types of individuals run a greater risk of experiencing negative consequences of home confinement. Therefore, it is important to bring psychological treatment and support groups to these individuals as soon as possible. In relation to this particular issue, mobile health-related applications have been suggested as an alternative to provide easy, immediate access to the treatments that are needed [29]. However, not all available health-related mobile apps have been developed on the basis of scientific tenets [30] following available guides, and undergoing usability tests and efficacy studies [31]. Therefore, additional studies are needed to improve the development of data-based health-related mobile apps and facilitate a wider implementation and use among healthcare professionals and patients. The data also showed that 5% of the participants reported having bad overall health and 4% having a bad quality of life. Sex and age showed a statistically significant association with physical health, with males and older participants (i.e., over 40 years old) reporting worse overall health. In addition, most participants (66%) reported pain problems, which were chronic in half of the cases. The percentage of individuals with pain in this sample was higher than in studies conducted in the early stages of home confinement. For example, Wang et al. [20] found that 10% of the participants in their study experienced headaches and 8% musculoskeletal pain. In Spain, Rodríguez-Rey et al. [22] found that 43% of adults in their sample had headaches and 19% musculoskeletal pain. Al-Hashel and Ismail [32], in a survey of 1018 adults suffering from migraines conducted between 15 July and 30 July 2020, found a 60% increase in the frequency of pain episodes, and a transition from acute to chronic pain in 10% of the cases. If these results are found to be valid, they would suggest that confinement contributes to worsening patients’ pain. However, additional research is needed to clarify whether this is indeed the case, and, more importantly, to explain why, how, and for whom this is so. In addition, research on the association between increased prevalence and intensity of pain with worsening mental health is warranted.

Participants also reported mild levels of fatigue. Interestingly, the data showed that 75% of participants exercised. This finding differs from the findings of other studies, which found that exercise and physical activity were reduced during lockdown [33,34]. Nonetheless, in a recent sample of Belgian adults, those younger than 55 who performed low-level physical activity before lockdown reported an increase in exercise during the home-confinement period [35]. One explanation for the divergence in the findings is that these previous studies were conducted during the early stages of confinement, when exercise outside of the home was not possible. In Spain, during the late stages of the state of emergency, people were allowed to leave their homes to exercise, and some individuals took advantage of that opportunity. However, it is unclear whether this higher level of exercise is related to a relaxation in the confinement conditions or to the fact that the participants habitually did physical exercise before lockdown. In addition, we do not have information about the amount or intensity of the exercise reported by this sample. Generally speaking, physical exercise is positively associated with better mental health [36,37,38]. In this study, most participants (81%) reported good to excellent overall health. It is unclear whether this was partly due to physical exercise, and additional longitudinal studies to evaluate this are warranted. Nevertheless, the promotion of healthy behaviors, such as practicing physical exercise, could have a significant impact on both mental and physical health.

Regarding the socio-economic impact of the COVID-19 lockdown, 39% of the sample reported a reduction in household income. This finding is higher than that described by Esteban-Gonzalo et al. [23] who reported that 26% of their study’s participants experienced a reduction in income. Such a reduction could also impact on many other individual domains. For example, it could increase worry and decrease physical and mental health. In summary, the COVID-19 home-confinement period impacted several areas of daily life, particularly the “motivation to work”, “satisfaction with life”, and “happiness” domains, with females reporting the greatest impact. Longitudinal studies to evaluate how these variables are related and to predict physical and mental health and well-being during confinement are needed.

Finally, nearly half of the participants (47%) reported wanting more information about the pandemic. This is in line with the results reported by Rodríguez-Rey et al. [22]. Importantly, their data showed that those participants who wanted more information about the pandemic also reported worse mental health [22]. Therefore, research should be undertaken on how to improve communication strategies across stakeholders (e.g., mass media, health-care professionals, government officials, and the general population), taking into account the information sources that are regularly used. The association between the communication of information and mental health should also be studied.

This study has some limitations that should be acknowledged. First, the participants consisted of individuals that were motivated to participate in this study. Although we did our best to promote and provide information about the launch of the survey, most of the respondents were from Catalonia, although we did not make any attempt for this. Therefore, we do not know the extent to which the sample is representative of the general population of adults living in Spain. However, the social distancing measures implemented during the lockdown in Spain were the same for all the regions of the country. In addition, only 4% of the respondents were over 65. Most of our efforts to promote participation in the survey were through social media, and this may be in part responsible for the limited number of elderly participants. However, lockdown reduced the number of other alternatives by which to conduct this type of research. Moreover, it is unclear if by using some other procedure (e.g., telephone interviews) we might have been able to increase the participation of this age group. Second, this was a cross-sectional study. Therefore, it is not possible to draw conclusions regarding the causal impact of the associations found. Longitudinal studies to evaluate possible causal associations are thus warranted.

## 5. Conclusions

Despite the study’s limitations, this study has yielded new information on the impact of the COVID-19 lockdown on the mental, physical, and socio-economic status of adults living in Spain. The findings showed that the negative effects on the three domains were present in the late stages of the state of emergency (when restrictions started to lessen in Spain). Importantly, female, younger, single, and less educated participants reported a greater impact on their mental health resulting from the restrictions applied due to COVID-19. This finding is similar to those reported in other studies with different samples (see the systematic review carried out by Xiong et al. [8]) and thus demonstrates the need for strategies to prevent these problems from worsening and new ones from developing in the late stages of a state of emergency.

The findings from this study can now be used to aid in the development of these programs. From a psychosocial perspective, they should be aimed at reducing the levels of anxiety, depression, and stress, providing support to individuals who have previous chronic conditions, and training them in the use of adaptive coping skills (e.g., staying active). These effects could be achieved, for example, by promoting social interactions while keeping some physical distance, such as using social networks; or developing online psychological support groups in which people could interact and share their needs and worries, learn relaxation techniques and better, more adaptable ways of coping with the pandemic; and establish healthy routines related to physical exercise and sleep. The entire population could benefit from these programs, although special attention should be paid to those most vulnerable, which seem to be younger (<40 years old), female, single, and less educated individuals.

## Figures and Tables

**Table 1:** Descriptive data for the study sample (N = 544).

	Mean (SD)	N	%
Age	38.57 (14.99)		
18–24 years old		147	27
25–40 years old		154	28
41–65 years old		224	41
Over 65 years old		19	4
Gender			
Female		423	78
Male		121	22
Education			
No studies or some basic studies		17	3
Secondary studies		207	38
University studies		320	59
Marital status			
Single		218	40
Married or living with a partner		284	52
Divorced		39	7
Widow/widower		4	1
Monthly family income			
<950€		16	4
951€–1900€		106	29
1901€–3800€		151	41
3801€–7600€		69	19
7601€–15,200€		6	2
>15,201€		18	5

**Table 2:** Descriptor statistics for mental health-related variables (N = 460).

Domain	N (%)	Mean (SD)	Range
Anxiety symptoms		5.61 (2.26)	0–42
Normal	314 (69)		
Mild	32 (7)		
Moderate	66 (14)		
Severe	17 (4)		
Extremely severe	30 (6)		
Depression symptoms		21.29 (8.12)	0–40
Normal	322 (70)		
Mild	47 (10)		
Moderate	45 (10)		
Severe	25 (5)		
Extremely severe	21 (5)		
Stress symptoms		17.74 (5.03)	0–42
Normal	312 (68)		
Mild	46 (10)		
Moderate	51 (11)		
Severe	31 (7)		
Extremely severe	20 (4)		
Worries			
One’s own health and of close people		7.99 (2.54)	0–10
Future		7.71 (2.53)	0–10
Being supportive with others		7.01 (2.69)	0–10
Own education or the education of children		6.75 (3.57)	0–10
Interpersonal relationships		6.61 (3.09)	0–10
Household’s income		6.59 (3.36)	0–10
Work		6.08 (3.47)	0–10
Others being supportive with you		5.83 (3.14)	0–10
Paying the mortgage/rent		5.43 (3.81)	0–10
Paying bills		5.38 (3.76)	0–10
Food		5.03 (3.23)	0–10

**Table 3:** Logistic regression analyses explaining mental health.

Variables	B	Wald	*p*-Value	OR (95%CI)
**Dependent variable: Anxiety**				
Age				
18–24 years (reference)		8.37	0.039 *	1
25–40 years	−0.70	4.76	0.029 *	0.50 (0.27–0.93)
41–65 years	−0.98	7.49	0.006 **	0.38 (0.19–0.76)
>65 years	−1.42	2.75	0.097	0.24 (0.05–1.30)
Gender	1.07	12.60	<0.001 ***	2.92 (1.62–5.27)
Academic level	−0.08	0.14	0.712	0.92 (0.61–1.40)
Marital status	−0.11	0.21	0.649	0.90 (0.57–1.41)
Change in household income	0.21	1.81	0.178	1.23 (0.91–1.66)
**Dependent variable: Depression**				
Age	−0.10	0.94	0.332	0.99 (0.97–1.01)
Gender	0.66	5.40	0.020 *	1.93 (1.11–3.35)
Academic level				
No studies (reference)		5.75	0.056	1
Secondary studies	−0.58	0.92	0.339	0.56 (0.17–1.83)
University studies	−1.02	3.04	0.081	0.36 (0.11–1.34)
Marital status:				
Single (reference)		8.11	0.044 *	1
Married or living with a partner	−0.74	6.36	0.012 *	0.48 (0.27–0.85)
Divorced	−1.25	4.75	0.029 *	0.29 (0.09–0.88)
Widow/widower	−0.39	1.73	0.772	0.68 (0.50–9.30)
Change in household income	0.20	2.05	0.189	1.22 (0.91–1.65)
**Dependent variable: Stress**				
Age	−0.02	4.78	0.029 *	0.98 (0.96–1.00)
Gender	1.18	15.19	<0.001 **	3.25 (1.80–5.89)
Academic level	−0.18	0.87	0.352	0.83 (0.57–1.22)
Marital status	−0.09	0.15	0.703	0.92 (0.59–1.42)
Change in household income	0.22	2.21	0.137	1.25 (0.93–1.66)

* *p* < 0.05; ** *p* < 0.01 *** *p* < 0.001

**Table 4:** Descriptor statistics for physical health-related variables (N= 544).

Domain	N (%)	Mean (SD)	Range
Overall health		2.67 (1.05)	1–5
Excellent	74 (13)		
Very good	167 (31)		
Good	195 (36)		
Reasonable	82 (15)		
Bad	27 (5)		
Quality of life		2.76 (0.96)	1–5
Excellent	45 (8)		
Very good	171 (32)		
Good	217 (40)		
Reasonable	84 (16)		
Bad	24 (4)		
Fatigue		3.17 (2.52)	0–10
Pain in the last 3 months	360 (66)		
Acute pain	188 (52)		
Chronic pain	172 (48)		
Pain location			
Back	232 (64)		
Head	211 (59)		
Neck	140 (39)		
Shoulders	102 (28)		
Legs	94 (26)		
Feet	64 (18)		
Hands	59 (16)		
Belly/pelvis	59 (16)		
Bottom/hips	55 (15)		
Chest/breast	46 (13)		
Arms	46 (13)		
Other locations	5 (1)		
Physical exercise	409 (75)		
Walking	239 (58)		
Biking	108 (26)		
Running	85 (21)		
Dumbbells	83 (20)		
Aerobics and cardio at home	61 (15)		
Yoga/Pilates	37 (9)		
Ball sports	18 (4)		

**Table 5:** Descriptor statistics for socio-economic impact variables (N = 493).

Domain	N (%)	Mean (SD)	Range
Change in household income			
Dropped > 50%	45 (11)		
Dropped < 50%	118 (28)		
Stayed equal	246 (58)		
Increased < 50%	15 (3)		
Increased > 50%	0		
COVID-19 interference			
Life in general		8.08 (2.06)	0–10
Interpersonal relationships		7.47 (2.60)	0–10
Work		7.10 (3.37)	0–10
Motivation to work		6.25 (3.48)	0–10
Satisfaction with life		6.24 (3.04)	0–10
Happiness		5.73 (3.02)	0–10

**Table 6:** Descriptor statistics for the variables about management of the pandemic (N = 493).

Domain	N (%)	Mean (SD)	Range
Enough information about pandemic			
Yes	264 (53)		
No	229 (47)		
Government management	167 (73)		
Treatment of COVID-19	132 (58)		
Effects of COVID-19	124 (54)		
Duration and characteristics confinement	66 (29)		
Others	22 (10)		
Improvements in government management			
No	43 (9)		
Yes	450 (91)		
Performing mass COVID tests	328 (73)		
Advancing confinement	323 (72)		
Improving political coordination	280 (62)		
Advancing use of masks	256 (57)		
Allowing businesses to open	22 (5)		
Others	43 (10)		
Trust in the Spanish health system		3.34 (1.03)	1–5

## Data Availability

The data presented in this study are available on request from the corresponding author.

## Supplementary Materials

The following are available online at https://www.mdpi.com/article/10.3390/ijerph19020854/s1, Table S1: Mean comparisons for mental health-related variables: anxiety, depression and stress (N = 460); Table S2: Mean comparisons for mental health-related variables: worries (N = 460); Table S3: Mean comparisons for physical health-related variables (N = 544) and Table S4: Mean comparisons for socio-economic variables: COVID-19 interference (N = 493).
