# Supplementary material for: Mental, Physical and Socio-Economic Status of Adults Living in Spain during the Late Stages of the State of Emergency Caused by COVID-19
Source: Int J Environ Res Public Health. 2022 Jan 13;19(2):854. doi: 10.3390/ijerph19020854 (PMC8775499; doi:10.3390/ijerph19020854)
Supplement: Supplementary file 1 [file ijerph-19-00854-s001.zip › ijerph-1483717-supplementary.pdf]

Table S1: Mean comparisons for mental health-related variables: anxiety, depression and stress (N=460).

Domain	Anxiety			Depression			Stress		
	**Mean (SD)**	**t/F***	**p**	**Mean (SD)**	**t/F***	**p**	**Mean (SD)**	**t/F***	**p**
**Gender**									
Females	6.52 (7.12)	4.27	**<.001**	8.25 (8.57)	2.60	**<.01**	13.34 (9.54)	4.69	**<.001**
Males	3.58 (5.87)			5.83 (7.83)			8.78 (8.50)		
**Age**									
18 – 24	7.74 (7.46) [a]	5.71	**<.001**	11.23 (9.81) [a]	12.48	**<.001**	15.59 (11.21) [a]	9.17	**<.001**
25 – 40	6.31 (7.40) [b]			8.20 (8.28) [b]			13.01 (9.40) [b]		
41 – 65	4.69 (5.98) [a]			5.57 (7.21) [ab]			10.19 (8.06) [ab]		
Over 65	3.14 (3.30) [ab]			4.80 (4.52) [a]			9.07 (5.39) [a]		
**Studies**									

No studies or elemental	5.69 (4.75)	7.16	**<.001**	8.62 (5.12)	3.59	**.028**	11.23 (6.03)	3.38	**.035**
Secondary studies	7.02 87.82) [a]			9.62 (9.32) [a]			13.83 (10.19) [a]		
University studies	5.20 (6.47) [a]			6.55 (7.87) [a]			11.47 (9.12) [a]		
**Marital status**									
Single	7.19 (7.76) [a]	3.89	**.009**	10.64 (9.80) [a]	13.23	**<.001**	14.20 (10.72) [a]	4.11	**.007**
Married or living with a partner	5.19 (6.56) [a]			6.08 (7.12) [a]			11.28 (8.65) [a]		
Divorced	3.94 (4.56) [a]			4.11 (4.99) [a]			10.00 (7.06) [a]		
Widow/widower	4.67 (5.03)			7.33 (7.02)			11.33 (6.43)		

Note: Highlighted p were significant at .05, .01 or .001. * t –test were computed for comparison between gender; ANOVAs were computed for comparisons between more than 2 groups. Superscript letters refer to categories between which there are significant differences.

Table S2: Mean comparisons for mental health-related variables: worries (N=460).

	Gender Mean (SD)			Age Mean (SD)					Education Mean (SD)				Marital status Mean (SD)				
**Worries**	**Female**	**Male**	**t (p)**	**18 - 24**	**25 - 40**	**41 - 65**	**>65**	**F (p)**	**Elementary**	**Secondary**	**University**	**F (p)**	**Single**	**Married living with a partner**	**Divorced**	**Widow / widower**	**F (p)**
One's own health and of close people	8.20 (2.37)	7.30 (2.94)	2.94 **(.004)**	7.85 (2.57)	8.27 (2.54)	7.94 (2.58)	7.06 (1.48)	1.45 (.227)	8.79 (1.48)	8.13 (2.62)	7.86 (2.53)	1.37 (.256)	7.70 (2.88)	8.25 (2.18)	7.72 (2.88)	8.33 (1.53)	1.93 (.123)
Future	7.86 (2.53)	7.21 (2.48)	2.37 (**.018**)	8.14 (2.26)[a]	7.68 (2.57)	7.64 (2.50)	5.38 (3.46)[a]	6.02 (**<.001**)	8.21 (2.97)	8.17 (2.22)[a]	7.40 (2.65)[a]	5.63 (**.004**)	7.75 (2.54)	7.68 (2.51)	7.64 (2.76)	8.00 (2.00)	.06 (.982)
Being supportive with others	7.21 (2.68)	6.34 (2.62)	3.03 (**.003**)	6.77 (2.79)	7.01 (2.76)	7.11 (2.56)	7.69 (2.70)	.79 (.503)	7.71 (3.45)	7.19 (2.54)	6.87 (2.73)	1.27 (.282)	6.57 (2.97)[a]	7.37 (2.36)[a]	6.89 (2.94)	7.67 (1.53)	3.45 **(.017)**
Own education or the education of children	6.87 (3.57)	6.35 (3.60)	1.35 (.178)	7.37 (3.04)	6.46 (3.78)	6.74 (3.61)	4.44 (4.27)	3.92 (**.009**)	7.00 (3.94)	7.56 (3.12)[a]	6.23 (3.74)[a]	7.99 (**<.001**)	6.37 (3.65)	7.21 (3.32)	5.42 (4.42)[a]	9.00 (1.00)[a]	4.25 **(.006)**

Interpersonal	6.80	5.96	2.51	6.88	7.03	6.31	4.56	4.31	6.36	7.01	6.38	2.36	6.54	6.74	6.19	5.67	.49
relationships	(3.07)	(3.11)	(**.012**)	(2.99)	(2.98)	(3.14)	(3.29)	(**.005**)	(3.61)	(3.039	(3.09)	(.095)	(3.18)	(3.00)	(3.36)	(2.08)	(.688)
Household's	6.76	5.99	2.13	7.09	7.17	6.08	3.88	7.78	7.64	7.42	6.03	10.75	6.85	6.46	6.31	4.67	.92
income	(3.36)	(3.30)	(**.033**)	(3.06)^{a}	(3.17)^{b}	(3.52)^{ab}	(3.18)^{ab}	(**<.001**)	(3.43)	(3.03)^{a}	(3.44)^{a}	(**<.001**)	(3.30)	(3.37)	(3.55)	(4.73)	(.429)
Work	6.20	5.66	1.42	5.79	6.98	5.96	1.88	12.21	6.29	6.06	6.08	.03	5.83	4.29	6.22	2.67	1.64
	(3.50)	(3.35)	(.156)	(3.43)^{a}	(3.17)^{ab}	(3.43)^{bc}	(3.34)^{abc}	(**<.001**)	(4.50)	(3.64)	(3.31)	(.973)	(3.43)	(3.49)	(3.34)	(4.62)	(.178)
Others being supportive with you	6.03 (3.15)	5.15	2.61	5.83	5.89	5.80	5.69	.03	6.31	6.14	5.62	1.74	5.66	6.01	5.42	7.00	.83
		(3.02)	(**.009**)	(3.17)	(3.22)	(3.05)	(3.59)	(.992)	(3.59)	(3.04)	(3.17)	(.176)	(3.20)	(3.06)	(3.39)	(1.73)	(.479)
Paying the	5.63	4.75	2.12	5.32	6.36	5.05	2.94	6.03	6.64	6.31	4.83	9.53	5.28	5.50	5.92	4.00	.47
mortgage/rent	(3.85)	(3.65)	(**.034**)	(3.70)	(3.75)^{a}	(3.83)^{a}	(3.30)^{a}	(**<.001**)	(4.18)	(3.76)^{a}	(3.72)^{a}	(**<.001**)	(3.80)	(3.81)	(3.91)	(5.29)	(.429)
Paying bills	5.58	4.70	2.17	5.20	6.40	4.96	3.19	6.48	6.36	6.20	4.83	8.25	5.32	5.43	5.50	4.33	.12
	(3.79)	(3.58)	(**.031**)	(3.67)^{a}	(3.64)^{ab}	(3.78)^{b}	(3.25)^{b}	(**<.001**)	(4.14)	(3.79)^{a}	(3.63)^{a}	(**<.001**)	(3.76)	(3.73)	(3.95)	(5.13)	(.948)
Food	5.13	4.67	1.31	4.57	5.45	5.04	4.81	1.71	6.43	5.49	4.67	5.10	4.66	5.24	5.36	7.33	1.88
	(3.28)	(3.04)	(.193)	(3.18)	(3.29)	(3.20)	(3.12)	(.164)	(3.03)	(3.13)^{a}	(3.25)^{a}	(**.006**)	(3.16)	(3.22)	(3.60)	(1.53)	(.132)

Note: Highlighted p were significant at .05, .01 or .001. Superscript letters refer to categories between which there are significant differences.

Table S3: Mean comparisons for physical health-related variables (N=544).

Domain	Fatigue			Overall health			Quality of life		
	**Mean (SD)**	**t/F***	**p**	**Mean (SD)**	**t/F***	**p**	**Mean (SD)**	**t/F***	**p**
**Gender**									
Females	3.39 (2.51)	4.01	**<.001**	2.77 (1.05)	4.05	**<.001**	2.82 (.94)		
Males	2.36 (2.42)			2.34 (.98)			2.54 (1.01)	2.82	**.005**
**Age**									
18 – 24	3.22 (2.31)	.96	.410	2.46 (1.05)	2.52	.057	2.64 (1.01)	.30	.828
25 – 40	3.16 (2.46)			2.58 (.97)			2.81 (.98)		
41 – 65	3.21 (2.70)			2.81 (1.06)			2.80 (.92)		
Over 65	2.21 (2.39)			3.32 (1.00)			2.72 (.96)		
**Studies**									

No studies or elemental	2.82 (2.92)	.19	.830	2.94 (1.14)	2.43	.089	3.00 (.61)	2.99	.051
Secondary studies	3.21 (2.59)			2.77 (1.09)			2.87 (1.03)		
University studies	3.16 (2.46)			2.59 (1.01)			2.68 (.93)		
**Marital status**									
Single	3.22 (2.40)	.62	.602	2.52 (1.02)	2.52	.057	2.71 (1.02)	.30	.828
Married or living with a partner	3.14 (2.59)			2.77 (1.07)			2.78 (.94)		
Divorced	3.18 (2.70)			2.74 (1.02)			284 (.89)		
Widow/widower	1.50 (3.00)			2.75 (.50)			2.75 (.50)		

Note: Highlighted p were significant at .05, .01 or .001. * t –test were computed for comparison between gender; ANOVAs were computed for comparisons between more than 2 groups.

Table S4: Mean comparisons for socio-economic variables: COVID-19 interference (N=493).

	Gender Mean (SD)			Age Mean (SD)					Education Mean (SD)				Marital status Mean (SD)				
**COVID-19 interference areas**	**Female**	**Male**	**t (p)**	**18 - 24**	**25 - 40**	**41 - 65**	**>65**	**F (p)**	**Elementary**	**Secondary**	**University**	**F (p)**	**Single**	**Married living with a partner**	**Divorced**	**Widow / widower**	**F (p)**
Life in general	8.17	7.78	1.75	7.83	8.24	8.33	5.56	10.42	6.43	7.96	8.24	5.74	8.24	8.26	8.06	8.00	1.37
	(2.11)	(1.88)	(.080)	(2.19)[a]	(1.83)[b]	(1.92)[c]	(2.90)[abc]	(**<.001**)	(2.98)	(2.22)	(1.88)	(**.003**)	(1.88)	(2.02)	(2.11)	(1.00)	(.251)
Interpersonal	7.53	7.27	.92	7.11	7.40	7.89	5.88	4.77	5.71	7.42	7.59	3.57	7.59	7.71	7.64	6.00	2.03
relationships	(2.68)	(2.35)	(.359)	(2.74)[a]	(2.70)	(2.28)[a]	(3.52)	(**.003**)	(3.99)	(2.71)	(2.43)	(**.029**)	(2.43)	(2.57)	(2.39)	(4.36)	(.109)
Work	7.19	6.80	1.07	6.72	7.57	7.32	3.19	9.41	4.29	6.90	7.35	6.17	7.35	7.26	7.22	3.00	1.87
	(3.38)	(3.34)	(.286)	(3.44)[a]	(3.15)[b]	(3.18)[c]	(4.37)[abc]	(**<.001**)	(5.14)	(3.60)	(3.05)	(**.002**)	(3.05)	(3.39)	(3.16)	(5.20)	(.133)
Motivation to work	6.48	5.42	2.85	6.97	6.73	5.75	2.38	11.47	5.29	6.45	6.18	.90	6.18	5.79	5.92	2.67	5.49
	(3.48)	(3.37)	**(.005)**	(3.04)[a]	(3.43)[b]	(3.52)[a]	(3.61)[ab]	(**<.001**)	(4.67)	(3.51)	(3.40)	(.407)	(3.40)[a]	(3.63)[a]	(3.51)	(4.62)	(**.001**)

Satisfaction with life	6.43	5.57	2.62	6.27	6.45	6.18	5.06	1.06	6.21	6.42	6.14	.47	6.14	6.35	5.47	7.00	.93
	(3.06)	(2.89)	(**.009)**	(3.15)	(2.86)	(3.07)	(3.19)	(.367)	(3.47)	(3.16)	(2.94)	(.626)	(2.94)	(2.92)	(3.68)	(1.00)	(.425)
Happiness	5.98	4.83	3.56	5.73	5.99	5.67	4.13	1.88	5.36	5.90	5.64	.54	5.64	5.75	5.36	5.67	.19
	(3.02)	(2.87)	(**<.001**)	(3.04)	(2.92)	(3.09)	(2.53)	(.132)	(3.67)	(3.21)	(2.87)	(.585)	(2.87)	(2.91)	(3.63)	(2.08)	(.901)

Note: Highlighted p were significant at .05, .01 or .001. Superscript letters refer to categories between which there are significant differences.
